# Measurement properties of oral health assessments for non-dental healthcare professionals in older people: a systematic review

**DOI:** 10.1186/s12877-019-1349-y

**Published:** 2020-01-03

**Authors:** Babette Everaars, Linet F. Weening-Verbree, Katarina Jerković-Ćosić, Linda Schoonmade, Nienke Bleijenberg, Niek J. de Wit, Geert J. M. G. van der Heijden

**Affiliations:** 1 University of Applied Sciences Utrecht, Research Group Innovations in Preventive Care, Heidelberglaan 7, 3512 CS Utrecht, The Netherlands; 2 Department of Social Dentistry, Academic Centre for Dentistry Amsterdam (ACTA), University of Amsterdam and VU University, Gustav Mahlerlaan 3004, 1081LA Amsterdam, The Netherlands; 3 Hanze University of Applied Sciences Groningen, Center of Dentistry and Oral Hygiene, University Medical Center Groningen, University of Groningen (RUG), A. Deusinglaan 1, 9713 AV Groningen, The Netherlands; 4 University of Applied Sciences Utrecht, Research group innovations in Preventive Care, Heidelberglaan 7, 3512 CS Utrecht, The Netherlands; 5 Medical Library, VU University Amsterdam, De Boelelaan 1117, P.O. Box 7057, 1007 MB Amsterdam, The Netherlands; 6 Utrecht University, University Medical Center Utrecht, Julius Center for Health Sciences and Primary Care, Huispost Str.6.131, 3508 GA Utrecht, The Netherlands

**Keywords:** Oral health assessment, Non-dental healthcare professional, Older people, Oral health

## Abstract

**Background:**

Regular inspection of the oral cavity is required for prevention, early diagnosis and risk reduction of oral- and general health-related problems. Assessments to inspect the oral cavity have been designed for non-dental healthcare professionals, like nurses. The purpose of this systematic review was to evaluate the content and the measurement properties of oral health assessments for use by non-dental healthcare professionals in assessing older peoples’ oral health, in order to provide recommendations for practice, policy, and research.

**Methods:**

A systematic search in PubMed, EMBASE.com, and Cinahl (via Ebsco) has been performed. Search terms referring to ‘oral health assessments^’^**,** ‘non-dental healthcare professionals’ and ‘older people (60+)’ were used. Two reviewers individually performed title/abstract, and full-text screening for eligibility. The included studies have investigated at least one measurement property (validity/reliability) and were evaluated on their methodological quality using “The Consensus-based Standards for the selection of health Measurement Instruments” (COSMIN) checklist. The measurement properties were then scored using quality criteria (positive/negative/indeterminate).

**Results:**

Out of 879 hits, 18 studies were included in this review. Five studies showed good methodological quality on at least one measurement property and 14 studies showed poor methodological quality on some of their measurement properties. None of the studies assessed all measurement properties of the COSMIN. In total eight oral health assessments were found: the Revised Oral Assessment Guide (ROAG); the Minimum Data Set (MDS), with oral health component; the Oral Health Assessment Tool (OHAT); The Holistic Reliable Oral Assessment Tool (THROAT); Dental Hygiene Registration (DHR); Mucosal Plaque Score (MPS); The Brief Oral Health Screening Examination (BOHSE) and the Oral Assessment Sheet (OAS). Most frequently assessed items were: lips, mucosa membrane, tongue, gums, teeth, denture, saliva, and oral hygiene.

**Conclusion:**

Taken into account the scarce evidence of the proposed assessments, the OHAT and ROAG are most complete in their included oral health items and are of best methodological quality in combination with positive quality criteria on their measurement properties. Non-dental healthcare professionals, policymakers and researchers should be aware of the methodological limitations of the available oral health assessments and realize that the quality of the measurement properties remains uncertain.

## Background

Nowadays, in Western countries more older people retain all or a major part of their natural teeth which brings along new challenges for the oral healthcare system. Highly complicated restorations (e.g. crowns, bridges, implants) make it more difficult to perform adequate oral self-care, especially in frail older people [1], and as such may result in (oral) health-related complications [2, 3].

Oral health problems like pain, abscesses, difficulties with eating and chewing may have a significant impact on older peoples’ self-esteem, well-being, social life, and quality of life [4, 5]. At the same time, oral problems like periodontitis are associated with for example cardiovascular diseases, diabetes and pneumonia [6, 7]. Therefore, prevention and early diagnosis of oral diseases are important for the risk reduction of developing further problems with oral and general health.

Oral health prevention requires regular inspection of the oral cavity. Such inspections are traditionally performed by the dentist during preventive treatment sessions in dental practice. However, several barriers to seeking oral health care may contribute to a decrease in oral inspections. A review from Kiyak et al. (2005) concluded that barriers in seeking oral care in older people are depending on age, ethnicity, income, availability of dental insurances, type of residence (urban vs. rural), physical access and general health. Moreover, they concluded that attitude and psychosocial factors could contribute to older peoples’ oral healthcare-seeking behavior. Since (frail) older people seek less frequently dental care, the role of non-dental care professionals gained importance in contributing to screen and triage oral health problems [8–11].

Over twenty years, several oral health assessments have been developed for use by non-dental healthcare professionals like nurses and caregivers. For example, the Oral Health Assessment Tool (OHAT), the Revised Oral Assessment Guide (ROAG), The Holistic Reliable Oral Assessment Tool (THROAT), and comparable assessments have been developed for inspection and triage the oral cavity of older people [10, 12]. Such assessments may serve non-dental healthcare professionals, for example in the context of assessing oral health in older people. Moreover, specific oral assessments have been developed for cancer patients [13]. However, since this target group suffers from specific oral health issues like Mucositis, their oral healthcare demand differs from general older people and was not the focus of this review.

Available oral health assessment as reported in the literature may differ in their approach and they are described as tools, instruments, guides, and sheets for oral cavity inspection or triage. In this review, we use the generic term oral health assessment for all of the approaches that aim to inspect the oral cavity of older people. Earlier studies reported that oral health assessments in practice should be: easy and simple to use, inexpensive, and only require basic equipment [10, 14]. Moreover, for evidence-based care decisions, the measurement properties of such (oral health) assessments are considered crucial and therefore should be tested. The measurement properties are divided into three domains [15, 16]:

- Validity, i.e. construct validity: align with the theoretical notion of oral health; content validity: include all items considered relevant by all stakeholders; criterion validity: correlates with a reference;
- Reliability, i.e. similar results are obtained for repeated measurements;
- Responsiveness, i.e. change over time is detected.

Chalmers et al. (2005) performed a systematic review on oral health assessments for use by nurses and caregivers of older people with dementia [10]. They concluded that there is a lack of validated and reliable tools for oral cavity inspection by non-dental healthcare professionals. Since then, new oral health assessments have been developed. Some of these were tested on their validity and reliability [17–19], while others were not [13, 20, 21]. To date, an overview of these assessments and their measurement properties has not been published.

### Objective

The purpose of this systematic review was to evaluate the content and the measurement properties of oral health assessments for use by non-dental healthcare professionals in assessing older peoples’ oral health, in order to provide recommendations for practice, policy, and research.

## Methodology

### Study design and strategy

To identify all relevant publications, systematic searches were performed in the bibliographic databases PubMed, EMBASE.com, and Cinahl (via Ebsco) from inception to 13 November 2017. Search terms included indexed terms from MeSH in PubMed, EMtree in EMBASE.com, Cinahl headings in Cinahl as well as free text terms. Search terms referring to ‘oral health assessments^’^ were used in combination with search terms comprising ‘non-dental healthcare professionals’ and ‘older people’ (60+). Duplicate studies were excluded. The full search strategies for all databases can be found in Additional file 1 (Search strategies for databases). Reference lists of included studies were screened for additional relevant studies (cross-reference check).

### Selection process

Two reviewers (BE and LWV) independently screened all potentially relevant titles and abstracts for eligibility. The selection process was performed using Covidence, a Cochrane online technology platform, to fulfill this procedure at distance [22]. If necessary, the full-text article was checked for the eligibility criteria. Differences in judgment were resolved through a consensus procedure. Studies were included if they met the following criteria: (i) full text available of the original article; (ii) include oral health assessments for oral cavity inspection of older people (60+) developed for use by non-dental healthcare professionals; (iii) report original investigative data on one or more measurement properties. Moreover, they should fulfill the criteria as defined by The Consensus-based Standards for the selection of health Measurement Instruments (COSMIN) for systematic reviews: www.database.cosmin.nl [23].

Studies were excluded if they concerned: (i) publications in other languages than English; (ii) oral health assessments developed for dental professionals; (ii) oral health-related quality of life instruments; (iii) oral screening instruments based only on questionnaires; and (iiii) oral health assessments exclusively developed for patients with cancer or another specific illnesses.

### General information of the included studies

To give an overview of the included studies, information has been extracted on: authors, publication year, study design, investigated measurement property, type of non-dental healthcare professional, specification of the older people population, oral health assessment (and their items assessed), rating scale of the assessment and duration of the assessment. Data extraction was performed on all included studies.

### Assessment of the methodological quality of the included studies per measurement property

**Fig. 1:**
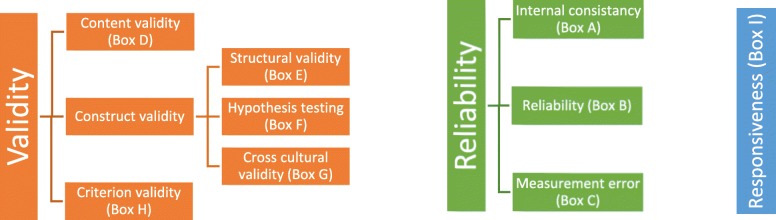
Items and boxes as used by the COSMIN checklist rated on a four-point scale: excellent, good, fair & poor

When validity and reliability of an assessment tool are investigated in a study of good methodological quality, the results can be used in research or daily care. However, when the methodological quality of a study is inadequate, the results of the study cannot be trusted and the quality remains unclear [16]. Therefore, to assess the methodological quality of the included studies, The COSMIN 4-point scale checklist has been used [24]. This checklist is a tool for the assessment of the methodological quality of studies examining measurement properties and has shown good inter-rater agreement and user-friendliness [19]. The COSMIN checklist evaluates three main measurement properties: 1. Validity, 2. Reliability, and 3.Responsiveness (Fig. 1), which are further divided into nine measurement properties (Box A-I). A visualization of how these measurement properties are related is shown in Fig. 1. Within the COSMIN a separate score is assigned for the methodological quality of each of the nine measurement properties in a study. Depending on the measurement property that has been evaluated, multiple scores for the methodological quality can be assigned and the score can differ per measurement property. For example, the methodological quality investigating the content validity can be good, while at the same time, the reliability assessment was performed in a small sample size and therefore of poor methodological quality. Depending on the measurement property, the COSMIN checklist contains a minimum of 5 and a maximum of 18 questions to evaluate the methodological quality [24]. Scores per question were rated on a nominal scale (excellent, good, fair, poor). To determine the methodological quality per property ‘The worst score counts’ criterion is used, meaning that the lowest score on a question within one measurement property determines the methodological quality score. For the full assessments of all measurement properties, we refer to the original COSMIN guideline [24]. A definition of each measurement properties is given in Table 1 under the column ‘*description*’. Definitions are based on Terwee et al. (2007) and slightly modified in terminology to fit the content of our study.

**Table 1:** Definitions of the measurement properties and their quality criteria

Measurement property			Description ^a^	Quality criteria for measurement properties ^b^
Validity	Content validity		To which degree the construct assesses whether the items are relevant for the construct to be measured	+: The target population considers all items in the instrument to be relevant AND to be complete
				?: No target population involvement
				-: The target population considers the items of the instrument irrelevant OR incomplete
	Construct validity	Structural validity	To which degree the scores of an instrument are an adequate reflection of the dimensionality	+: Factors should explain at least 50% of the variance
				?: Explained variance not mentioned
				-: Factors explain < 50% of the variance
		Hypothesizes testing	To which extent the scores of the instrument are consistent with the theoretically derived hypotheses	+: Correlation with an instrument measuring the same construct ≥ 0.50 or at least 75% of the results are in accordance with the hypotheses AND correlation with related constructs is higher than with unrelated constructs
				?: Solely correlations determined with unrelated constructs
				-: Correlations with an instrument measuring the same construct <0.50 OR <75% of the results are in accordance with the hypotheses OR correlation with related constructs is lower than with unrelated constructs
		Cross-cultural validity	To which extend the items are an adequate reflection of the original version after translation or culturally adaptation.	+: no important DIF between language versions
				?: DIF not assessed
				-: Important DIF found between language versions
	Criterion validity		To what degree the scores of the instrument are an adequate reflection of a ‘gold standard’. The gold standard should fit the purpose of the assessed instrument.	+: Convincing arguments that gold standard is ‘’gold” AND correlations with gold standard ≥0.70
				?: No convincing argument that gold standard is ‘’gold” OR doubtful design or method
				-: Despite adequate design and method, correlation is < 0.70
Reliability	Reliability		The proportion of the total variance in the measurements which is because of ‘’true” differences among patients	+: ICC/weighted kappa ≥ 0.70 OR Pearson’s r ≥ 0.80
				?: Neither ICC/weighted kappa, nor Pearson’s r determined
				-: ICC/weighted kappa <0.70 OR Pearson’s r < 0.80
	Internal consistency		The extent to which items in a sub(scale) are inter correlated, thus measuring the same construct	+: Cronbach’s ^α^ (s) ≥ 0.70
				?: Cronbach’s ^α^ not determined
				-: Cronbach’s ^α^ < 0.70
	Measurement error		The systematic and random error of a patient’s score that is not attributed to true changes in the construct to be measured	+:MIC <SDC OR MIC outside the LOA OR convincing arguments that agreement is acceptable
				?: Doubtful design or method OR MIC not defined AND no convincing arguments that agreement is acceptable
				-: MIC≥ SDC OR MIC equals or inside LOA, despite adequate design and method
Responsiveness			The ability of the instrument to detect change over time	+: Correlation with an instrument measuring the same construct ≥ 0.50 OR at least 75% of the results are in accordance with the hypotheses OR AUC ≥ 0.70 AND correlation with related constructs is higher than with unrelated constructs
				?: Solely correlations determined with unrelated constructs
				-: Correlation with an instrument measuring the same construct <0.50 OR <75% of the results are in accordance with the hypotheses or AUC <0.70 OR correlation with related constructs is lower than with unrelated constructs.

*DIF* Differential item functioning, *MIC* minimal important change, *SDC* Smallest detectable change, *LOA* Limits of agreement, *ICC* Intra Class Correlation

+= positive rating; ?= indeterminate rating; -= negative rating

^a^Descriptions of the measurement properties are based on Terwee et al (2007)

^b^To fit the content of oral health assessments, we combined the quality criteria as used by Weldam et al. (2013) & Terwee (2007)

Two raters (BE & LWV) independently determined the overall methodological quality per property. A disagreement between the raters was resolved via a consensus meeting. A third reviewer (KJ) was consulted when an agreement was still not reached.

### Quality criteria for the measurement properties on oral health assessments

When measurement properties were of excellent, good or fair methodological quality, an assessment of the *quality* of the measurement properties has been performed. Measurement properties of poor methodological quality were excluded for further quality assessment of this specific measurement property. The scores for quality of measurement property were: positive (+), negative (−) or indeterminate (?). See the column ‘Quality criteria for measurement properties’ in Table 1 for the definitions.

## Results

### Search results

**Fig. 2:**
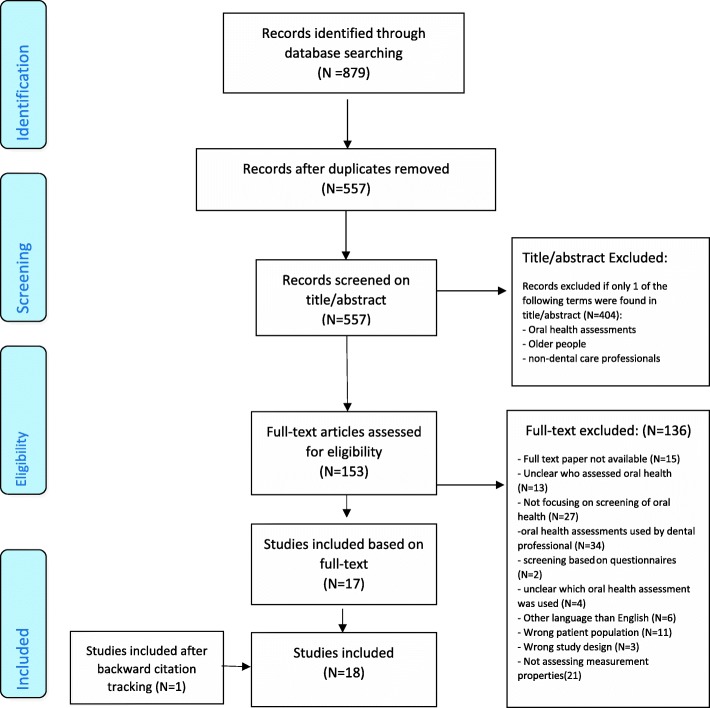
Flowchart of in- and excluded studies

The literature search generated a total of 879 references: 395 in PubMed, 393 in EMBASE.com and 91 in Cinahl. After removing duplicates, 557 references remained. Four hundred four studies were removed based on the screening of the title and the abstract. The flowchart of the search and selection process is presented in Fig. 2. After screening the full-text, 136 studies were removed based on the presented in-and exclusion criteria. One article which met the in-and exclusion criteria was added after reviewing the reference lists of included articles. Reasons for exclusion full-text articles are described in Fig. 2.

### Included studies

In total, 18 studies describing eight different oral health assessments were included for analysis: (1) The Revised Oral Assessment Guide (ROAG); (2) the Minimum Data Set (MDS), with oral health component; (3) the Oral Health Assessment Tool (OHAT); (4) The Holistic Reliable Oral Assessment Tool (THROAT); (5) Dental Hygiene Registration (DHR); (6) Mucosal Plaque Score (MPS); (7) the Brief Oral Health Screening Examination (BOHSE), and (8) the Oral Assessment Sheet (OAS). Table 2 gives an overview of the included studies and their investigated oral health assessments. Most non-dental healthcare professionals involved were nurses, sub-classified as Registered Nurse (RN), Licensed Vocational Nurse (LVN), Clinical Nurse (CN) or Licensed Practical Nurse (LPN). In the study of Simpelaere et al. (2016), speech pathologists were included [38]. The population on which the oral health assessment was used was heterogeneous and consisted of rehabilitation residents, nursing home residents, hospitalized older people, community-dwelling older people and older people with mental problems (Table 2).

**Table 2:** Data-extraction table for the included studies

	Authors	Publication year	Study design	Investigated measurement property	Type of non-dental healthcare professional using assessment	Patient population	Oral health assessment	Rating scale	Duration of assessment
1	Andersson et al. [18]	2002	Cross-sectional observational	Inter-rater reliability	RN	older people in rehabilitation ward	ROAG	3 point scale on 8 items	Unknown
2	Andersson et al. [25]	2002	Cross-sectional observational	Content validity	RN	Geriatric rehabilitation patients	ROAG	3 point scale on 8 items	Unknown
3	Arvidson-Bufano et al. [26]	1996	Cross-sectional observational	Inter-rater reliability	RN and LPN	Nursing home residents	MDS-RAI (section M) and RAP summary	2 Point scale on 7 items	3–4 min
4	Blank et al. [27]	1996	Cross-sectional observational	Inter-rater reliability	RN and LPN	Nursing home residents	MDS-RAI (section M) and RAP summary	2 Point scale on 7 items	Unknown
5	Chalmers et al. [17]	2005	Prognostic follow-up	Content validity Criterion validity Intra-rater reliability Inter-rater reliability Test-retest reliability	PCA, RN, Enrolled Nurses and NA	Residents from residential facilities	OHAT	3 point scale on 8 items	Mean: 7.8 min
6	Cohen-Mansfield et al. [28]	2002	Cross-sectional observational study	Inter-rater reliability	Geriatricians	Nursing home residents with Dementia	MDS- mouth pain and inflamed gums	8 items on 2 point scale	Unknown
7	Dickinson et al. [19]	2001	Cross-sectional study	Content validity Intra-rater reliability Inter-rater reliability	Stroke specialist nurse, staff nurses, student nurse	Older medically Ill patients	THROAT	4 point scale on 9 items	Unknown
8	Fjeld et al. [29]	2016	Prognostic follow-up	Content validity Criterion validity Inter-rater reliability	Clinical nurse	Nursing home residents	DHR	3 point scale on two items	Less than 1 minute
9	Hanne et al. [30]	2012	Cross-sectional	Cross-cultural validity	Nurses	Acute medical ward residents (mean age 76.5)	ROAG	3 point scale on 8 items	Unknown
10	Hawes et al. [31]	1995	Cross-sectional	Inter-rater reliability	LN	Nursing home residents	MDS	Unclear	Unknown
11	Henriksen et al. [32]	1999	Cross-sectional	Intra-rater reliability Inter-rater reliability	Medical Nurse	older people with mental disabilities	MPS	4 point scale on 2 items	2–4 min
12	Kayser-Jones et al. [33]	1995	Cross-sectional	Inter-rater reliability Test-retest reliability	RN, LVN, CNA	Nursing home residents	BOHSE	3 point scale on 10 items	Mean time RNs, LVNS, CNAs: 7.4, 7.9 and 8.7 min
13	Lin et al. [34]	1999	Cross-sectional	Criterion validity Inter-rater reliability	LN and CNA	LTC residents with Alzheimer	BOHSE	3 point scale on 10 items	Unknown
14	Morris et al. [35]	1997	Cross-sectional	Inter-rater reliability	Nurses	Community-dwelling older people with home care	MDS-HC	Unclear	Unknown
15	Paulsson et al. [36]	2008	Prospective	Criterion validity	Nurses	Patients on medical ward (mean age 67)	ROAG	3 point scale on 8 items	Unknown
16	Riberio et al. [37]	2014	Cross-sectional	Cross-cultural validity Criterion validity Intra-rater reliability	CHW	Community-dwelling older people	ROAG	3 point scale on 8 items	11 min
17	Simpelaere et al. [38]	2016	Cross-sectional with two- week follow-up for test-retest	Intra-rater reliability Inter-rater reliability Test-retest reliability	Speech Pathologists	Acute geriatric department/hospitalized, residential care settings (assisted living and nursing homes)	OHAT	3 point scale on 8 items	Mean time: 2.45 min
18	Yanagisawa et al. [39]	2017	Cross-sectional	Internal consistency Inter-rater reliability	Caregivers	Institutionalized older people	OAS	3 point scale on 9 items	Unknown

Non-dental healthcare abbreviations: *RN* Registered Nurse, *LVN* Licensed Vocational Nurse, *CN* Clinical Nurse, *LPN* Licensed Practical Nurse, *DDS* Doctoral Dental Surgery, *DNS* Director of Nursing, *CHW* Community health workers, *NA* Nurse assistant, *PCA* Personal Care Attendants.

Oral health assessment abbreviations: *ROAG* The Revised Oral Assessment Guide, (2) *MDS-RAI/RAP* the Minimum Data Set-Resident Assessment Instrument/ Resident Assessment Protocol, *OHAT* with oral health component, (3) the Oral Health Assessment Tool, (4) *THROAT* The Holistic Reliable Oral Assessment Tool, (5) *DHR* Dental Hygiene Registration, (6) *MPS* Mucosal Plaque Score, (7) *BOHSE* the Brief Oral Health Screening Examination and the *OAS* Oral Assessment Sheet

### The methodological quality of the included studies per measurement property

None of the studies assessed all measurement properties included in the COSMIN checklist. Chalmers et al. (2005) investigated the most (*N* = 5) measurement properties of the OHAT (Table 2). In total, five studies showed good methodological quality on at least one measurement property and 14 studies showed poor methodological quality on some of their measurement properties. An overview of the reasons for poor methodological quality is shown in Table 3. Below, the results on the methodological quality per measurement property will be described. The following measurement properties were not investigated by any of the included studies: Measurement error (box C), Structural validity (box E), Hypothesis testing (box F) and Responsiveness (box I).

**Table 3:** Reasons for scoring poor methodological quality on the measurement property for assessing oral health per study

Study	Assessment	Measurement property	Reason for poor methodological quality
Andersson et al. (2002b) [25]	ROAG	Content validity	- Target population not involved - Not assessed if all items together comprehensively reflect the construct to be measured
Arvidson-Bufano et al. (1996) [26]	MDS-RAI	Inter-rater reliability	- Small sample size - Only percent agreement calculated
Blank et al. (1996) [27]	MDS-RAI	Inter-rater reliability	- Unclear how many patients the dentist assessed - Only percent agreement is calculated - Other important methodological flaws in design or execution of study
Chalmers et al. (2005) [10]	OHAT	Content validity Criterion Validity Test-retest	- Target population not involved - Not assessed if all items together comprehensively reflect the construct to be measured - Small sample size - No ICC or correlation calculated
Cohen-Mansfield et al. (2002) [28]	MDS	Inter-rater reliability	- Small sample size - No ICC or correlations calculated - Other important methodological flaws in design or execution of study
Dickinson et al. (2001) [19]	THROAT	Content validity	- Target population not involved
Fjeld et al. (2017) [29]	DHR	Content validity	- Target population not involved
Hanne et al. (2012) [30]	ROAG	Cross-cultural validity	- Only forward translation
Hawes et al. (1995) [31]	MDS	Inter-rater reliability	- Only percent agreement is calculated
Henriksen et al. (1999) [32]	MPS	Intra-rater reliability Inter-rater reliability	- Small sample size
Kayser-Jones et al. (1995) [33]	BOHSE	Content validity	- Target population not involved
Paulsson et al. (2008) [36]	ROAG	Criterion validity	- Other important methodological flaws in design or execution of study - Correlations or AUC not calculated - Sensitivity and specificity not calculated
Simpelaere et al. (2016) [38]	OHAT	Intra-rater reliability	- Small sample size - Only percent agreement is calculated
Yanagisawa et al. (2017) [39]	OAS	Criterion-validity	- No factor analysis performed and no reference to another study

### The methodological quality of the measurement property validity

Nine out of the 18 included studies investigated the domain validity of the oral health assessments (Table 4).

**Table 4:** Methodological quality of the measurement property “validity” by the COSMIN and quality criteria of the measurement properties per assessment

Assessment	Study	Validity					
		Content validity		Cross-cultural validity		Criterion Validity	
		M	Q	M	Q	M	Q
ROAG	Andersson et al. (2002b) [25]	Poor	N.A.				
	Hanne et al. (2012) [30]			Poor	N.A.		
	Paulsson et al. (2008) [36]					Poor	N.A.
	Ribeiro et al. (2014) [37]			Fair	?	Good^a^	? (Sens: 0.17-0.80) (Spec: 0.69-0.98)
OHAT	Chalmers et al. (2005) [17]	Poor	N.A.			Poor	N.A.
THROAT	Dickinson et al. (2001) [19]	Poor	N.A.				
DHR	Fjeld et al. (2017) [29]	Poor	N.A.			Fair	+ (r(s) = 0.78)
BOHSE	Kayser-Jones et al. (1995) [33]	Poor	N.A.				
	Lin et al. (1999) [34]					Good^a^	- (r: 0.351-0.578)

M = Assessment of methodological quality: “excellent”, “good”, “fair”, “poor”’ by COSMIN. Q = criteria for measurement properties; + = positive rating;? = indeterminate rating; − = negative rating.

^a^For criterion validity, a non-dental healthcare professional was the index-rater, a dentist was used as reference-rater

*N.A*. Not applicable was reported for the quality criteria when an article had poor methodological quality.

Of those, all five studies that assessed content validity, scored poor on their methodological quality, mainly because the patient population was not involved in developing the oral health assessment and studies did not assess if the items comprehensively reflect the construct (i.e. “oral health”) to be measured [19, 25, 29, 33, 40] (see Table 3). Two studies assessed cross-cultural validity. The ROAG was translated in Portuguese by Riberio et al. (2014) using multiple forward translations and one backward translation [37]. Hanne et al. (2012) only conducted forward translation into Danish and scored therefore poor on the methodological quality [30] (Table 3).

Criterion validity was assessed by five studies on the ROAG, OHAT, DHR, and BOHSE. Chalmers et al. (2005) and Paulsson et al. (2008) scored poor on their methodological quality on this property (Table 3). Riberio et al. (2014) assessed the ROAG on criterion validity with a dentist considered as “gold standard” (reference-rater) and had good methodological quality [37]. Fjeld et al. (2017), investigated the criterion validity on the DHR and Lin et al. (1999) on the BOHSE [29, 34]. They scored fair and good on the methodological quality on the measurement property respectively (Table 4).

The studies investigating the MDS, MPS, and OAS were not assessed on any validity items [26–28, 31, 32, 35, 39].

### The methodological quality of the measurement property reliability

For this study, the reliability was divided into intra-rater reliability, inter-rater reliability, and test-retest to assess the methodological quality. Internal consistency was only investigated by the study of Yanagisawa et al. (2017) but was of poor methodological quality [39] (Table 3).

### Intra-rater reliability

The intra-rater reliability was investigated for the ROAG, OHAT, THROAT, MPS, and DHR. Good methodological quality of the intra-rater reliability assessment was performed for the ROAG and THROAT by Ribeiro et al. (2014) and Dickinson et al. (2001) respectively [19, 37] (Table 5). The studies of Chalmers et al. (2005) and Simpelaere et al. (2016) investigated the intra-rater reliability for the OHAT [17, 38]. Chalmers et al. (2005) only reported unweighted kappas and was therefore of fair methodological quality.

**Table 5:** Methodological quality of the measurement property “reliability” by the COSMIN and quality criteria of the measurement properties per assessment

Assessment	Study	Reliability								
		Internal-consistency		Intra-rater reliability		Inter-rater reliability		Test-retest reliability		Raters
		M	Q	M	Q	M	Q	M	Q	
ROAG	Andersson et al. (2002a) [18]					Good^a^	?/− (κ/κ^w^: 0.45-0.84)^b^			Nurse/Dental hygienist
	Ribeiro et al. (2014) [37]			Good	+/− (κ^w^: 0.38-0.88)					Community health workers
MDS	Arvidson-Bufano et al. (1996) [28]					Poor^a^	N.A.			Nurse/Dentist
	Blank et al. (1996) [27]					Poor^a^	N.A.			Nurse/Dentist
	Cohen-Mansfield (2002) [28]					Poor^a^	N.A.			Geriatricians/Dentist
	Hawes et al. (1995) [31]					Poor	N.A.			Nurses
MDS-HC	Morris et al. (1997) [35]					Good	+/− (κ^w^: 0.57-0.7)			Nurses
OHAT	Chalmers et al. (2005) [17]			Fair	+ (ICC = 0.78) ? (κ: 0.51-0.80)^b^	Fair	+ (ICC = 0.74) ? (κ: 0.48-0.80)^b^	Poor	N.A.	Nurses
	Simpelaere et al. (2016) [38]			Poor	N.A.	Fair	+ (ICC = 0.96) ? (κ: 0.83-1.00)	Fair	+ (ICC = 0.81 & 0.78) ? (κ: 0.14-0.91)	Speech pathologists
THROAT	Dickinson et al.(2001) [19]			Good	+/− (κ^w^: 0-0.96)	Good^a^	+/− (κ^w^: 0.46-0.97)			Dental hygienist,/ stroke specialist nurse and staff Nurse
DHR	Fjeld et al. (2017) [29]			Fair	+ (κ: 0.7-0.8)	Fair^a^	? (κ: 0.4-0.8)			Dental hygienist and Nurse
MPS	Henriksen et al. (1999) [32]			Poor	N.A.	Poor^a^	N.A.			Dentist, 2 Dental Hygienist, and Nurse
BOHSE	Kayser-Jones et al. (1995) [33]					Fair^a^	-(r: 0.4-0.68) ? (κ: -0.02-0.82)^b^	Fair	+/− (r: 0.79-0.88)	Dentist and Nurses
	Lin et al. (1999) [34]					Fair^a^	? (κ: -0.018-0.519)^b^			Dentist and Nurses
OAS	Yanagisawa et al. (2017) [39]	Poor	N.A			Fair	? (κ: 0.25-0.90) +/- (ICC: 0.54-0.98)			Dental professionals and care workers

M = Assessment of methodological quality: “excellent”, “good”, “fair”, “poor” by COSMIN. Q = criteria for measurement properties; + = positive rating;? = indeterminate rating; − = negative rating.

^a^ Inter-rater reliability measurements have been performed by two different professions.

^b^Only kappas are reported instead of percent agreement because this reflects better methodological quality according to the COSMIN criteria

*N.A*. Not applicable was reported for the quality criteria when an article had poor methodological quality.

Simpelaere et al. (2016) and Henriksen et al. (1999) scored poor methodological quality for this property (Table 3). Fjeld et al. (2017) scored fair methodological quality on this measurement property.

### Inter-rater reliability

Inter-rater reliability was assessed for all oral health assessments in 14 included studies. Inter-rater reliability was investigated between several professions: nurses, speech pathologists or a dental professional with a non-dental healthcare professional (Table 5). Only three studies scored good on the methodological quality: Andersson et al. (2002), testing the ROAG, Morris et al., testing the MDS-HC and Dickinson et al. (2001), testing the THROAT [18, 19, 35]. The MDS was assessed on inter-rater reliability by all five studies on MDS. However, the quality was rated poor for four of them because of the low quality of the statistical method and small sample size (Table 3) [26–28, 31].

Studies investigating the OHAT, DHR, BOHSE, and OAS scored fair on methodological quality on the inter-rater reliability mainly because they reported unweighted kappas for ordinal scores [17, 29, 33, 39]. The study of Henriksen et al. (1999), showed poor methodological quality (Table 3) [32].

### Test-retest reliability

Simpelaere et al. (2016) and Chalmers et al. (2005) investigated the stability of the OHAT by a test-retest. Chalmers et al. (2005) did not report correlations over time and therefore scored poor on the methodological quality (Table 3). Kayser-Jones et al. (1995) (BOSHE) also looked at test-retest reliability. The methodological quality was fair because of the moderate sample size and reported unweighted kappas for the ordinal score.

### Characteristics of individual oral health assessments and the quality assessment of their measurement properties

Overall, the oral health assessments include 18 items in the oral cavity. The most frequently assessed items are lips, mucosa membrane, tongue, gums, teeth, denture, saliva, and oral hygiene (Table 6). The assessments of each item can differ. For example the item “Lips”: some assessments assess it by color and moistness while others look at swelling and bleeding (Table 6).

**Table 6:** Items which are assessed by the different oral health assessments

	ROAG^a^	MDS^b^	OHAT^b/c^	THROAT^a^	DHR	MPS	BOHSE^d^	OAS
1. Mucosa membrane	**X**	**X**	**X**	**X**		**X**	**X**	**X**
Color/Rash	X	X	X	X		X	X	
Moistness	X		X	X			X	
Swelling/glazing/granulations/Hyperplasia	X		X	X		X	X	
Bleeding	X		X	X		X	X	
Ulcers / Spots (under dentures)	X	X	X	X		X	X	X
2. Gums	**X**	**X**	**X**	**X**			**X**	
Color	X		X	X			X	
Moistness			X	X				
Swelling/glazing	X		X	X			X	
Bleeding	X		X	X			X	
Firmness	X						X	
Inflammation		X		X				
Ulceration/spots			X	X			X	
Loose teeth							X	
3. Teeth	**X**	**X**	**X**				**X**	
Decay/Cariës/Broken teeth	X	X	X				X	
Number of teeth			X				X	
Tooth erosion/wear			X					
4. Dentures	**X**	**X**	**X**				**X**	**X**
Broken parts	X		X				X	
Does the individual wear the dentures		X	X				X	
Fit of dentures/need for adhesive			X					X
Label on dentures			X					
Functionality								X
5. Lips	**X**		**X**	**X**			**X**	
Color	X		X	X			X	
Surface structure/Candida infection	X		X	X			X	
Moistness	X		X	X			X	
Ulceration	X		X	X			X	
Bleeding	X		X	X			X	
Swelling			X					
6. Tongue	**X**		**X**	**X**			**X**	**X**
Color	X		X	X			X	
Surface structure	X		X	X			X	
Moistness	X		X	X			X	
Ulceration/coating	X		X	X			X	X
Swelling	X		X					
Bleeding				X				
7. Saliva	**X**		**X**	**X**			**X**	**X**
Measured as friction/adherence of mouth mirror at buccal mucosa	X							
Amount/structure of saliva			X	X			X	X
Involvement of tissues			X				X	X
Experience of individual			X					
8. Palate				**X**			**X**	
Color				X			X	
Surface structure				X			X	
Moistness				X			X	
Ulceration				X			X	
Swelling							X	
Inflammation/bleeding				X			X	
9. Floor of mouth				**X**			**X**	
Color				X			X	
Surface structure				X			X	
Moistness				X			X	
Ulceration/coating				X			X	
Swelling							X	
Inflammation/bleeding				X			X	
10. Oral hygine (debris and plaque)	**X**		**X**	**X**	**X**	**X**	**X**	**X**
11. Referral to a dental professional	**X**		**X**					
12. Smell			**X**	**X**				**X**
13. Pairs in chewing position (amount)							**X**	**X**
14. Pain (physical signs and verbal signs)			**X**					
15. Voice (deep, rasping or painful)	**X**							
16. Ability to swallow (pain/inability to swallow)	**X**							
17. Functionality (mouth opening, tong thrusting)								**X**
18. Lymph nodes (enlargement and tenderness)							**X**	

a) The ROAG and THROAT assess the items “Teeth and Dentures”’, however, they actually look at plaque/debris and oral hygiene in this item. Therefore, we labeled these items as “Oral Hygiene”. b)The MDS and OHAT combine the items “Gums and Mucosa membrane” into one item. c) The OHAT does not have a separate item for smell. They included it in the item “Oral Hygiene”. d) The BOHSE combines the items “Mucosa Membrane”, “Floor of mouth” and “Palate” into one item.

If applicable, below the validity, intra−/inter-rater reliability and test-retest of the oral health assessments will be evaluated in their context and the quality assessment of the measurement property will be reported. No studies with acceptable methodological quality of any of the measurement properties were found for the MPS, so this assessment will not be discussed.

### ROAG

Andersson et al. (2002) conducted a study on the inter-rater reliability between a dental hygienist and a registered nurse [18]. The percent agreement was the lowest for teeth/dentures and tongue and the highest for swallowing and voice. Only weighted kappas (κ^w^) were reported on items that scored a minimum and maximum on the ordinal scale. For the items “voice”’ and “gums” no maximum score (score 3) was registered and therefore unweighted kappas (K) were reported instead of weighted Kappas. The quality assessment of the measurement property scored therefor? /−. The Kappas ranged from 0.45–0.84 with a mean of 0.59 (Table 5). The lowest kappas were found for voice (κ), teeth/dentures (κ^w^), tongue (κ^w^), and saliva (κ^w^) and the highest for swallowing (κ^w^).

Ribeiro et al. (2014) investigated the ROAG on validity and reliability in Portuguese [37]. Criterion validity was assessed with a dentist considered as “gold standard”(reference-rater). The measurement property was scored indeterminate (?) because sensitivity, specificity, and accuracy were reported. Sensitivity ranged from 0.17 for saliva to 1.0 for swallowing. Specificity ranged from 0.69 for teeth/dentures to 0.98 for saliva (Table 4). For intra-rater reliability for the community health workers (CHW’s), only weighted kappas were measured for the items with two or three levels of response: tongue, hygiene of teeth and dentures, and/or caries. They ranged from κ^w^ = 0.38 to κ^w^ = 0.88 and therefore scored +/− on the measurement property (Table 5). The lowest weighted kappa was found for teeth/dentures. Unweighted kappas were the lowest for saliva and the highest for voice, lips, and swallowing.

### MDS

The MDS was investigated by five different studies, however as described before, four of them had poor methodological quality and will not be evaluated in-depth. Morris et al. (1997), using the MDS-HC (for community-dwelling older people) reported overall weighted kappas between nurses for the oral health component ranging from κ^w^ = 0.57 to κ^w^ = 0.60. For MDS 2.0 (nursing homes) this was κ^w^ = 0.70. Because of the spread between weighted kappas, a +/− was scored for the quality criteria (see Table 5) [35].

### OHAT

Measurement properties of the OHAT were assessed by Chalmers et al. (2005) and Simpelaere et al. (2016). In the study of Chalmers et al. (2005), on individual item level, intra-rater reliability ranged from 74.4% agreement for oral cleanliness to 93.9% for dental pain and 96.6% for a referral to the dentist [17]. Unweighted kappas were moderate: 0.51–0.60 for lips, saliva, oral cleanliness and referral to the dentist. All other categories showed kappas ranging from 0.61–0.80, which indicates substantial agreement. The overall intraclass correlation coefficient on the total score was 0.78 and all results were statistically significant. The quality of measurement property was scored +/? because of its high Intra Class Correlation (ICC) and reported unweighted kappas (Table 5).

For the inter-rater reliability between nurses, percent agreement ranged from 72.6% for oral cleanliness to 92.6% for dental pain and 96.8% for the referral to the dentist. Unweighted kappas varied from 0.48–0.60 for lips, tongue, gums, saliva, oral cleanliness and referral to the dentist. The other items scored between 0.61 and 0.80, indicating substantial agreement for inter-rater reliability. The correlation coefficient for the inter-rater agreement on the total score was 0.74. All statistics were statistically significant. The quality of measurement property was scored +/? because of its high ICC and unweighted kappas were reported (Table 5).

Simpelaere et al. (2016) investigated the intra-, inter- and test-retest reliability in speech pathologists [38]. However, intra-rater reliability was of “poor” methodological quality as described earlier and will not be further described.

The inter-rater reliability was tested between three speech pathologists on 132 individuals. The ICC on the total score was 0.96 (95% CI 0.95–0.97) and scored therefore positive (+) on the quality criteria (Table 5). The individual items varied with a Fleiss kappa from 0.83 to 1.00. No weighted kappa was calculated, therefore an indeterminate (?) rating was given. For the test-retest, a second assessment was performed on 46 individuals after two weeks. The ICC for the two raters on the total score was 0.81 (95% CI 0.68–0.89) and 0.78 (95% CI 0.64–0.87). Kappas varied between 0.14 for dental pain and 0.91 for dentures and teeth. Another slight agreement was found for gums and tissues. Because of the reported unweighted kappas, and indeterminate (?) rating was scored (Table 5).

### Throat

For the intra-rater agreement investigated by Dickinson et al. (2001), the weighted kappas varied between κ^w^ = 0.69–0.96 for all items, except for the floor of the mouth and smell (κ^w^) = 0. For the total score, intra-rater reliability was good κ^w^ = 0.95 (95% CI 0.88–1.02) [19]. Because of the large spread between kappas, the measurement property scored +/− on the quality criteria (Table 4).

The Inter-rater assessment for the single items was performed between nurses and the dental hygienist reporting unweighted kappas of κ < 0.30 across the raters. Negative kappas were reported for teeth and smell. When raters were paired, the weighted kappas ranged from κ^w^ = 0.46-0.89, with the lowest values for teeth and dentures. Because of the spread between kappas a +/− was scored on the quality criteria.

A positive (+) rating for the inter-rater reliability on the total score was reported because weighted kappas were κ^w^ = 0.96 (95% CI 0.90–1.02) between a stroke specialist nurse and student nurse and κ^w^ = 0.97 (95% CI 0.92–1.02) between stroke specialist nurses and dental hygienist.

### DHR

Fjeld et al. (2017) developed and tested the DHR [29]. For criterion validity, a positive (+) rate was scored because correlations with their reported gold standards (Mucosal Plaque Index [32] and OHI-S [41]) was Rs = 0.78 and statistically significant (Table 4). For inter-rater reliability, the unweighted kappa between the dental hygienist and clinical nurse was κ = 0.4 (not statistically significant) and scored therefore indeterminate (?). Intra- and inter-rater reliability has also been evaluated on a series of videos. The inter-rater reliability was scored indeterminate (?) because the unweighted kappa for the dental hygienist was 0.7 and for the clinical nurse κ = 0.8 (Table 5).

### BOHSE

Lin et al. (1999) investigated the criterion validity using a dentist as “gold standard”(reference-rater) [34]. For criterion validity +/− was scored because the correlation coefficients varied between 0.351 and 0.578 for the dentist and the nurses (nurse and clinical nurse assistant (CNA)). However, correlation coefficients were lower than 0.70 and therefore they scored negative (−) on the quality criteria (Table 4).

Inter-rater reliability was also tested between the dentist and the nurses. An intermediate (?) score was given because only percent agreement and unweighted kappas were reported. The lowest percent agreements were found on the items lips, gums, natural teeth, and oral cleanliness: 60.7%, 37.5%, 60.7%, and 32.1% respectively. Kappas ranged from κ = 0.015 to κ = 0.519. The lowest kappas were reported for gums between the Doctor of Dental Surgery (DDS) and CNA and oral cleanliness between the DDS and the nurse. The highest kappa was reported for pairs of teeth in chewing position (Table 5). In addition, negative kappas were reported for: lymph nodes, lips, tongue and tissues/cheek and, the floor of the mouth.

In the study of Kayser-Jones et al. (1995) the inter-rater reliability on the total score was rated negative (−) because correlations varied between 0.40 (RN and CAN) and 0.68 (between the DDS and LVN) and were all statistically significant [33]. For the individual items, percent agreement ranged from 50.5–98.0. With the lowest values for oral cleanliness and the highest for lymph nodes. The unweighted kappas ranged from κ = 0.09 for the item tissues and κ = 0.82 for pairs in chewing position. Negative kappas were reported for lymph nodes. The individual items of the BOHSE scored indeterminate (?) because unweighted kappas were reported (Table 5).

The test-retest reliability was assessed on the total score by Kayser-Jones et al. (1995) for the DDS, RN, LVN, and CNA. The highest correlation was reported for the RN between time 1 and 2. The quality criteria scored +/− because statistically significant correlations varied between r = 0.79 and r = 0.88 between time 1 and 2 for different raters (Table 5).

### OAS

Yanagisawa et al. (2017) investigated the inter-rater reliability between dental professionals and carers before and after training [39]. Between dental professionals, the Fleiss’ kappa ranged from 0.49 to 0.83 and the ICC mean was 0.93. Kappa values were low for tongue coat, bad breath, and mouth opening.

The kappas between dental professionals and care workers ranged from 0.25–0.80 and were the highest for bad breath and tongue thrusting. After the training, the mean kappas increased to a mean of 0.72 and the ICC increased to 0.89, with the lowest values for the cleanliness of teeth and gums, bad breath and difficulty chewing. Indeterminate (?) score was reported because the unweighted kappas were reported and the ICC scored +/− because of the variance between the scores (Table 5).

## Discussion

With this systematic review, we evaluated eighteen studies, investigating eight oral health assessments for use by non-dental healthcare professionals to assess older peoples’ oral health, on their content and measurement properties in order to give recommendations for practice, policy and research.

Out of the eighteen included studies, only five of them scored good on the methodological quality of some of the measurement properties [18, 19, 34, 35, 37]. Overall, the OHAT has been most extensively investigated on its measurement properties with fair/good methodological quality and a positive(+)/indeterminate(?) quality assessment of the outcome. Similar results were found for the BOHSE (a prior version of OHAT) which was the most reliable and valid oral health assessment, according to the systematic review of Pearson and Chalmers in 2005 [10]. However, nurses concluded that the BOHSE was too long and complicated and therefore it has been simplified into the OHAT by Chalmers et al. (2005) [17, 33]. Three adaptations were made: 1. The category of lymph nodes and pairs of teeth in chewing position was eliminated; 2. The items tissue and gums were combined and 3. A category of behavioral problems and pain was added.

The ROAG, MDS, OHAT, THROAT, BOHSE, and OAS contain most items to inspect the oral cavity, varying between 6 and 12 items. The results of this review show the least agreement between raters on the items: oral hygiene, lips, saliva, and natural teeth. An explanation could be that non-dental healthcare professionals lack experience in assessing these items. Results from a focus group discussion from Chalmers (2005) support these findings; nurses felt less capable of assessing gums and tissues and natural teeth. Surprisingly, the nurses felt less capable of assessing the domain ‘pain’, which also showed the lowest kappa in the study of Simpeleare et al. (2016) between three speech pathologists.

Another remarkable result was the negative kappas in the study of Lin et al. (1999) for lymph nodes, lips, tongue, and tissues. In this study, they claim that a negative kappa for lymph nodes was found because the research population did not show enlarged lymph nodes during the study [34]. However, no explanation has been given for the other negative values. Literature states that a negative kappa can occur when the outcome is lower than expected or disagreement between two raters occurs [42]. However, more information on the context of the study is needed to give a reliable explanation. The study of Dickinson et al. (2001) reported negative kappas for the items teeth and smell. This study supports the explanation of too little variety between the scores [19]. Therefore they modified the THROAT by removing these items during further analysis.

As far as we know, this is the first systematic review that critically appraised the methodological quality of studies investigating the measurement properties of oral health assessments for use by non-dental healthcare professionals. When the methodological quality of the studies is lacking, the validity and reliability of the outcomes remain unclear [16]. Therefore, first, the methodological quality of the measurement property per study has been assessed. For this purpose, we used the COSMIN checklist with a 4-point scale [24]. Although recent updates of COSMIN are published, we chose to use the former version instead of the update. The updated COSMIN is specially developed for Patient-Reported Outcome Measures (PROMs), with a conditional step for good content validity for further assessment of other measurement properties [43], while the version of 2012 that we used focusses in a more general context on measurement properties of measurement instruments/assessments and therefore is better suited to our objective.

However, even the COSMIN version of 2012 lead to some discussion points in our study. Although developed for assessing measurement properties in a more general context, this version of COSMIN strongly emphasizes the involvement of the target population (patients) in developing a measurement instrument. As a result, content validity scored poor overall on the methodological quality in the included studies because none of the included studies involved patients in developing the oral health assessment [44]. Nevertheless, we doubt to what extent the input of patients should be highly rated in the development of an oral health assessment which is used by non-dental healthcare professionals. The input of experts and non-dental healthcare professionals, might, in this case, be more valuable. The included studies often consulted experts and non-dental healthcare professionals in the development of oral health assessments. Therefore, we think that the rating of poor methodological quality with the COSMIN on this item should be interpreted with reservations.

Regarding terminology, we noticed that “validity” and “reliability” are not used consistently in the included studies. We sometimes found mixed terminology for intra-rater reliability and test-retest reliability: Intra-rater reliability was described in the study, while a time interval of the second assessment was stated. Thus, in this case, test-retest would have been more appropriate.

In addition, comparisons between a dental professional and non-dental healthcare professionals were made in assessing the criterion validity in some studies, while other studies referred to this as inter-rater reliability. For inter-rater reliability, often a non-dental healthcare professional was compared to a dental care professional as the reference-rater. For criterion validity, the dental professional was referred to as the “gold standard”. The purpose of investigating the criterion validity is to compare the investigated instrument/assessments against a gold standard. However, no gold standard for oral health assessments exist. The OHAT and DHR were the only assessments in which the single items were assessed using several standardized criteria [17, 29]. However, these indices are not reported as gold standards. Since the aim of the oral health assessment is not to diagnose oral diseases but to screen and triage, we consider a dental professional as the expert in detecting oral problems and therefore we scored positive on the methodological quality of criterion validity when using a dental professional as “gold standard” (reference-rater).

Finally, a remark on the “worst score counts” method should be discussed: some studies scored good or excellent on a majority of the items, except for one single item, which resulted in a “poor” overall score. For example, the study of Chalmers et al. (2005) scored poor on the validity items because of the small sample size, while all other items scored good/excellent. This makes the method very strict in its overall score and this should be taken into account when referred to as “poor” methodological quality items.

### Recommendations for researchers, policymakers, and users

Based on our findings, we recommend more research on the measurement properties validity and reliability of the existing oral health assessments. This should be done in studies with good methodological quality as introduced by COSMIN. As a first step, there should be unanimity about the content of oral health assessments performed by non-dental healthcare professionals. Relevant stakeholders should determine which items assess a “healthy” versus “unhealthy” mouth. The FDI is working on a standardized set of oral health measures that could be used as background information and be adapted for this specific purpose (oral health assessment by non-dental healthcare professionals) [45]. In addition, when conducting research on the measurement properties, a proper distinction should be made between testing validity or reliability and the use of adequate statistical methods and analysis Furthermore, when investigating criterion validity, it is recommended to investigate the individual items of an oral health assessment using standardized criteria like the Mucosal Plaque Index and OHI-S, WHO oral lesions categories, Rise denture assessment and NIDR tooth status as conducted by Chalmers et al. (2005) and Fjeld et al. (2007) [17, 29]. Since research on validity and responsiveness requires “gold standards”, which are not available for all aspects of oral health, we recommend research on the standardization of oral health measures and the possibility to develop gold standards. Finally, when new oral health assessments for non-dental healthcare professionals are developed we recommend using the COSMIN guideline to minimize methodological flaws and develop highly reliable and valid oral health assessments [46].

Policymakers should take into account the level of education and proper training of the healthcare workers when implementing an oral health assessment. Training in using an oral health assessment might not be sufficient as there is a need for improvement of oral health knowledge of non-dental healthcare professionals in general [47]. Several studies concluded that non-dental healthcare professionals lack knowledge about oral health [1, 47–49]. A literature review concluded that educational programs delivered, regularly reinforced by a dental hygienist, and using several teaching formats were most effective in the improvement of oral health of patients [47]. Therefore, we recommend that a dentist or a dental hygienist is involved during the implementation of oral health assessments of older people for continues training and feedback to support non-dental healthcare professionals.

For non-dental healthcare professionals, we recommend taking into account the objective of assessing the oral cavity when choosing an oral health assessment. When screening, triage or decision for a referral to a dental professional is the main objective, the OHAT (prior BOHSE) and ROAG could be suitable. However, also other oral health assessments could be relevant when: (1) it is part of a general geriatric assessment (MPS); (2) the oral health assessment is for a specific patient group (THROAT); (3) only oral hygiene will be evaluated (DHR); or (4) the objective of an assessment is to give an indication of the oral health situation and set up an oral health care plan of patients in a specific setting (ROAG, OAS).

## Conclusion

In this systematic review, several oral health assessments have been evaluated on their measurement properties. Most studies suffer from methodological shortcomings (according to the COSMIN criteria). To increase the methodological quality of oral health assessments, and facilitate the investigation thereof in future research, standardization of oral health assessment is required.

Taken into account the scarce evidence of the proposed oral health assessments, the OHAT and ROAG are most complete in their included oral health items (including triage and referral to a dental professional when needed) and their studies are of best methodological quality in combination with a positive quality assessment on validity and reliability. Moreover, the OHAT has been most comprehensively investigated on its measurement properties. When choosing an oral health assessment, non-dental healthcare professionals should take such evidence into account. However, when using these oral health assessments one must realize that to date its evidence base is rather limited. Policymakers should be aware of the methodological limitations of the existing assessments when implementing them in healthcare and provide sufficient education for its users.

## Supplementary information

**Additional file 1.** Search strategies for databases. Search strategy per database

## Data Availability

Not applicable.

## Abbreviations

BOHSE: Brief Oral Health Screening Examination
CHW: Community Health Workers
CN: Clinical Nurse
COSMIN: The Consensus-based Standards for the selection of health Measurement Instruments
DDS: Doctor of Dental Surgery
DHR: Dental Hygiene Registration
DIF: Differential item functioning
DNS: Director of Nursing
ICC: Intra Class Correlation
κ: Kappa
κ^w^: Weighted Kappa
LOA: Limits Of Agreement
LPN: Licensed Practical Nurse
LVN: Licensed Vocational Nurse
MDS: Minimum Data Set
MIC: Minimal Important Change
MPS: Mucosal Plaque Score
NA: Nurse Assistant
OAS: Oral Assessment Sheet
OHAT: Oral Health Assessment Tool
PCA: Personal Care Attendants
PROM: Patient-Reported Outcome Measure
RN: Registered Nurse
ROAG: Revised Oral Assessment Guide
SDC: Smallest Detectable Change
THROAT: The Holistic Reliable Oral Assessment Tool

## References

[1] Everaars B, Jerkovic-Cosic K, van der GJ P, van der GJMG H. Probing problems and priorities in oral health (care) among community dwelling elderly in the Netherlands- a mixed method study. Int J Health Sci Res. 2015;5(9):415-29.

[2] Lee Kyung Hee, Plassman Brenda L., Pan Wei, Wu Bei (2015). Mediation Effect of Oral Hygiene on the Relationship Between Cognitive Function and Oral Health in Older Adults. Journal of Gerontological Nursing.

[3] Pretty Iain A. (2014). The life course, care pathways and elements of vulnerability. A picture of health needs in a vulnerable population. Gerodontology.

[4] Niesten D, van Mourik K, van der Sanden W (2012). The impact of having natural teeth on the QoL of frail dentulous older people. A qualitative study. BMC Public Health.

[5] The Gerontological Society of America. WHAT'S HOT Oral health: an essential element of healthy. Aging. 2017:20. https://www.geron.org/images/gsa/documents/oralhealth.pdf.

[6] Ottawa ON (2009). Optimal health for frail older adults: best practices along the continuum of care.

[7] Rautemaa R., Lauhio A., Cullinan M.P., Seymour G.J. (2007). Oral infections and systemic disease—an emerging problem in medicine. Clinical Microbiology and Infection.

[8] Niesten D. (2015). De invloed van kwetsbaarheid op mondzorggedrag en tandartsbezoek van ouderen. Nederlands Tijdschrift voor Tandheelkunde.

[9] Kiyak HA, Reichmuth M. Barriers to and enablers of older adults' use of dental services. J Dent Educ. 2005;69(9):975-86. 16141083

[10] Chalmers Jane M., Pearson A. (2005). A Systematic Review of Oral Health Assessment by Nurses and Carers for Residents with Dementia in Residential Care Facilities. Special Care in Dentistry.

[11] Rademakers L, Gorter RC. Aging and oral health care in the Netherlands. An explorative study. Ned Tijdschr Tandheelkd. 2008;115(10):527-32. 18979963

[12] RNAO (2008). Nursing best practice guideline Oral health: nursing assessment and interventions.

[13] Knoos M, Ostman M. Oral assessment guide--test of reliability and validity for patients receiving radiotherapy to the head and neck region. Eur J Cancer Care (Engl). 2010;19(1):53-60. 10.1111/j.1365-2354.2008.00958.x 19709166

[14] Rivett Donelle (2006). Compliance with best practice in oral health: implementing evidence in residential aged care. International Journal of Evidence-Based Healthcare.

[15] Mokkink Lidwine B., Terwee Caroline B., Patrick Donald L., Alonso Jordi, Stratford Paul W., Knol Dirk L., Bouter Lex M., de Vet Henrica C. W. (2010). The COSMIN checklist for assessing the methodological quality of studies on measurement properties of health status measurement instruments: an international Delphi study. Quality of Life Research.

[16] COSMIN Taxonomy of Measurement Properties. Available from: https://www.cosmin.nl/tools/cosmin-taxonomy-measurement-properties/. Accessed 19 June 2019.

[17] Chalmers JM, King PL, Spencer AJ, Wright FAC, Carter KD (2005). The Oral Health Assessment Tool — Validity and reliability. Australian Dental Journal.

[18] Andersson Pia, Hallberg Ingalill R., Renvert Stefan (2002). lnter-rater reliability of an oral assessment guide for elderly patients residing in a rehabilitation ward. Special Care in Dentistry.

[19] Dickinson H., Watkins C., Leathley M. (2001). The development of the THROAT: the holistic and reliable oral assessment tool. Clinical Effectiveness in Nursing.

[20] Peltola Petteri, Vehkalahti Miira M (2005). Chewing Ability of the Long-Term Hospitalized Elderly. Special Care in Dentistry.

[21] Munoz Nancy, Touger-Decker Riva, Byham-Gray Laura, Maillet Julie O'Sullivan (2009). Effect of an oral health assessment education program on nurses’ knowledge and patient care practices in skilled nursing facilities. Special Care in Dentistry.

[22] Covidence. 2016; Available at: https://www.covidence.org/. Accessed 9 Jan 2016.

[23] COSMIN database of systematic reviews of outcome measurement instruments. 2018. Available from: http://database.cosmin.nl/. Accessed 17 Apr 2018.

[24] Terwee Caroline B., Mokkink Lidwine B., Knol Dirk L., Ostelo Raymond W. J. G., Bouter Lex M., de Vet Henrica C. W. (2011). Rating the methodological quality in systematic reviews of studies on measurement properties: a scoring system for the COSMIN checklist. Quality of Life Research.

[25] Andersson Pia, Westergren Albert, Karlsson Siv, Rahm Hallberg Ingalill, Renvert Stefan (2002). Oral health and nutritional status in a group of geriatric rehabilitation patients. Scandinavian Journal of Caring Sciences.

[26] Arvidson-Bufano Ulla Britt, Blank Lawrence W., Yellowitz Janet A. (1996). Nurses' oral health assessments of nursing home residents pre- and post-training: A pilot study. Special Care in Dentistry.

[27] Blank Lawrence W., Arvidson-Bufano Ulla Britt, Yellowitz Janet A. (1996). The effect of nurses' background on performance of nursing home resident oral health assessments pre- and post-training. Special Care in Dentistry.

[28] Cohen-Mansfield Jiska, Lipson Steven (2002). The underdetection of pain of dental etiology in persons with dementia. American Journal of Alzheimer's Disease & Other Dementiasr.

[29] Fjeld Katrine Gahre, Eide Hilde, Mowe Morten, Hove Lene Hystad, Willumsen Tiril (2017). Dental hygiene registration: development, and reliability and validity testing of an assessment scale designed for nurses in institutions. Journal of Clinical Nursing.

[30] Hanne Konradsen, Ingelise Trosborg, Linda Christensen, Ulrich Pedersen P (2012). Oral status and the need for oral health care among patients hospitalised with acute medical conditions. Journal of Clinical Nursing.

[31] Hawes C., Morris J. N., Phillips C. D., Mor V., Fries B. E., Nonemaker S. (1995). Reliability Estimates for The Minimum Data Set for Nursing Home Resident Assessment and Care Screening (MDS). The Gerontologist.

[32] Henriksen Birgitte M., Ambjørnsen Eirik, Axéll Tony E. (1999). Evaluation of a mucosal-plaque index (MPS) designed to assess oral care in groups of elderly. Special Care in Dentistry.

[33] Kayser-Jones J., Bird W. F., Paul S. M., Long L., Schell E. S. (1995). An Instrument To Assess the Oral Health Status of Nursing Home Residents. The Gerontologist.

[34] Lin Christopher Y., Jones David B., Godwin Karen, Godwin R. Kenneth, Knebl Janice A., Niessen Linda (1999). Oral health assessment by nursing staff of Alzheimer's patients in a long-term-care facility. Special Care in Dentistry.

[35] Morris John N., Fries Brant E., Steel Knight, Ikegami Naoki, Bernabei Roberto, Carpenter G. Iain, Gilgen Ruedi, Hirdes John P., Topinková Eva (1997). Comprehensive Clinical Assessment in Community Setting: Applicability of the MDS-HC. Journal of the American Geriatrics Society.

[36] Paulsson G, Wardh I, Andersson P, Ohrn K. Comparison of oral health assessments between nursing staff and patients on medical wards. Eur J Cancer Care (Engl). 2008;17(1):49-55. 10.1111/j.1365-2354.2007.00802.x 18181891

[37] Ribeiro Marco Tulio F., Ferreira Raquel C., Vargas Andrea M.D., e Ferreira Efigênia Ferreira (2013). Validity and reproducibility of the revised oral assessment guide applied by community health workers. Gerodontology.

[38] Simpelaere Ingeborg S., Van Nuffelen Gwen, Vanderwegen Jan, Wouters Kristien, De Bodt Marc (2016). Oral health screening: feasibility and reliability of the oral health assessment tool as used by speech pathologists. International Dental Journal.

[39] Yanagisawa Shizuko, Nakano Masanori, Goto Takaharu, Yoshioka Masami, Shirayama Yasuhiko (2017). Development of an Oral Assessment Sheet for Evaluating Older Adults in Nursing Homes. Research in Gerontological Nursing.

[40] Wårdh Inger, Berggren Ulf, Andersson Lars, Sörensen Stefan (2002). Assessments of oral health care in dependent older persons in nursing facilities. Acta Odontologica Scandinavica.

[41] Greene John G., Vermillion Jack R. (1964). The Simplified Oral Hygiene Index. The Journal of the American Dental Association.

[42] McHugh ML. Interrater reliability: the kappa statistic. Biochem Med (Zagreb). 2012;22(3):276-82. PMC3900052 23092060

[43] Prinsen C. A. C., Mokkink L. B., Bouter L. M., Alonso J., Patrick D. L., de Vet H. C. W., Terwee C. B. (2018). COSMIN guideline for systematic reviews of patient-reported outcome measures. Quality of Life Research.

[44] Mokkink Lidwine B., Terwee Caroline B., Patrick Donald L., Alonso Jordi, Stratford Paul W., Knol Dirk L., Bouter Lex M., de Vet Henrica C.W. (2010). The COSMIN study reached international consensus on taxonomy, terminology, and definitions of measurement properties for health-related patient-reported outcomes. Journal of Clinical Epidemiology.

[45] FDI and ICHOM present Standard Set of Adult Oral Health Measures.; 2018. Available from: https://www.fdiworlddental.org/news/20180908/fdi-and-ichom-present-standard-set-of-adult-oral-health-measures. Accessed 2 Apr 2019.

[46] Terwee Caroline B., Bot Sandra D.M., de Boer Michael R., van der Windt Daniëlle A.W.M., Knol Dirk L., Dekker Joost, Bouter Lex M., de Vet Henrica C.W. (2007). Quality criteria were proposed for measurement properties of health status questionnaires. Journal of Clinical Epidemiology.

[47] Miegel Karen, Wachtel Tracey (2009). Improving the oral health of older people in long-term residential care: a review of the literature. International Journal of Older People Nursing.

[48] Wårdh Inger, Jonsson Margareta, Wikström Maude (2011). Attitudes to and knowledge about oral health care among nursing home personnel - an area in need of improvement. Gerodontology.

[49] Hollaar V, Maarel-Wierink v, Claar P v, Gert-Jan. RB, Elvers H, Cees BD, et al. Nursing staffs knowledge about and skills in providing oral hygiene care for patients with neurological disorders. J Oral Hyg Health. 2015;3(190). 10.4172/2332-0702.1000190.

