# Supplementary material for: Measurement properties of oral health assessments for non-dental healthcare professionals in older people: a systematic review
Source: BMC Geriatr. 2020 Jan 3;20:4. doi: 10.1186/s12877-019-1349-y (PMC6942417; doi:10.1186/s12877-019-1349-y)
Supplement: Supplementary file 1 — Additional file 1. Search strategies for databases. Search strategy per database [file 12877_2019_1349_MOESM1_ESM.docx]

Additional File 1. Search strategies for databases

**Search strategy in PubMed 13 November 2017 (read from bottom-up).**

| **Set** | **Search terms** | **Result** |
| --- | --- | --- |
| #4 | #1 AND #2 AND #3 | 395 |
| #3 | "Aged"[Mesh] OR "Aged, 80 and over"[Mesh] OR "Frail Elderly"[Mesh] OR "Geriatrics"[Mesh] OR "Geriatric Psychiatry"[Mesh] OR "Geriatric Nursing"[Mesh] OR "Geriatric Dentistry"[Mesh] OR "Dental Care for Aged"[Mesh] OR "Health Services for the Aged"[Mesh] OR "Alzheimer Disease"[Mesh] OR "Dementia"[Mesh] OR "Cognition Disorders"[Mesh] OR elder*[tw] OR eldest[tw] OR frail*[tw] OR geriatri*[tw] OR old age*[tw] OR oldest old*[tw] OR senior*[tw] OR senium[tw] OR very old*[tw] OR septuagenarian*[tw] OR octagenarian*[tw] OR octogenarian*[tw] OR nonagenarian*[tw] OR centarian*[tw] OR centenarian*[tw] OR supercentenarian*[tw] OR older people[tw] OR older subject*[tw] OR older patient*[tw] OR older age*[tw] OR older adult*[tw] OR older man[tw] OR older men[tw] OR older male[tw] OR older woman[tw] OR older women[tw] OR older female[tw] OR older population*[tw] OR older person*[tw] OR alzheimer*[tiab] OR demented[tiab] OR dementia[tiab] OR "cognitive decline"[tiab] OR "cognitive impairment"[tiab] OR nursing home*[tiab] OR assisted living facilit*[tiab] | 3082822 |
| #2 | "Nurses"[Mesh] OR "Caregivers"[Mesh] OR "Community Health Workers"[Mesh] OR "Physician Assistants"[Mesh] OR "Allied Health Occupations"[Mesh] OR "Geriatricians"[Mesh] OR "Speech-Language Pathology"[Mesh] OR "Nutritionists"[Mesh] OR nurs*[tiab] OR caregiver*[tiab] OR carer*[tiab] OR informal care*[tiab] OR community health worker*[tiab] OR physician assistant*[tiab] OR physician’s assistant*[tiab] OR physicians’ assistant*[tiab] OR doctor’s assistant*[tiab] OR doctor assistant*[tiab] OR non dental professional*[tiab] OR non dental staff[tiab] OR geriatrician*[tiab] OR gerontologist*[tiab] OR speech pathologist*[tiab] OR nutritionist*[tiab] OR dietician*[tiab] OR dietitian*[tiab] | 560482 |
| #1 | "Oral Hygiene Index"[Mesh] OR oral assessment*[tiab] OR (("Oral Hygiene"[Mesh] OR "Oral Health"[Mesh] OR mouth hygiene[tiab] OR oral health[tiab] OR oral care[tiab] OR oral hygiene[tiab] OR dental[tiab]) AND ("Needs Assessment"[Mesh] OR "Nursing Assessment"[Mesh] OR "Geriatric Assessment"[Mesh] OR assessment*[tiab] OR assessing[tiab] OR screening[tiab])) | 18598 |

**Search strategy in Embase.com 13 November 2017 (read from bottom-up).**

| **Set** | **Search terms** | **Result** |
| --- | --- | --- |
| #4 | #1 AND #2 AND #3 | 393 |
| #3 | 'aged'/exp OR 'geriatrics'/exp OR 'elderly care'/exp OR 'gerontopsychiatry'/exp OR 'geriatric nursing'/exp OR 'dementia'/de OR 'Alzheimer disease'/exp OR 'cognitive defect'/de OR elder*:de,ab,ti OR eldest:de,ab,ti OR frail*:de,ab,ti OR geriatri*:de,ab,ti OR (old NEXT/1 age*):de,ab,ti OR (oldest NEXT/1 old*):de,ab,ti OR senior*:de,ab,ti OR senium:de,ab,ti OR (very NEXT/1 old*):de,ab,ti OR septuagenarian*:de,ab,ti OR octagenarian*:de,ab,ti OR octogenarian*:de,ab,ti OR nonagenarian*:de,ab,ti OR centarian*:de,ab,ti OR centenarian*:de,ab,ti OR supercentenarian*:de,ab,ti OR 'older people':de,ab,ti OR (older NEXT/1 subject*):de,ab,ti OR (older NEXT/1 patient*):de,ab,ti OR (older NEXT/1 age*):de,ab,ti OR (older NEXT/1 adult*):de,ab,ti OR 'older man':de,ab,ti OR 'older men':de,ab,ti OR 'older male':de,ab,ti OR 'older woman':de,ab,ti OR 'older women':de,ab,ti OR 'older female':de,ab,ti OR (older NEXT/1 population*):de,ab,ti OR (older NEXT/1 person*):de,ab,ti OR alzheimer*:ab,ti OR demented:ab,ti OR dementia:ab,ti OR ‘cognitive decline’:ab,ti OR ‘cognitive impairment’:ab,ti OR ‘nursing home*’:ab,ti OR ‘assisted living facilit*’:ab,ti | 3182688 |
| #2 | 'nurse'/exp OR 'caregiver'/exp OR 'health auxiliary'/exp OR 'physician assistant'/exp OR 'speech language pathologist'/exp OR 'geriatrician'/exp OR 'dietitian'/exp OR nurs*:ab,ti OR caregiver*:ab,ti OR carer*:ab,ti OR ‘care giver*’:ab,ti OR ‘informal care*’:ab,ti OR ‘community health worker*’:ab,ti OR ‘physician* assistant*’:ab,ti OR ‘doctor* assistant*’:ab,ti OR ‘non-dental professional*’:ab,ti OR ‘non dental professional*’:ab,ti OR ‘non-dental staff’:ab,ti OR ‘non dental staff’:ab,ti OR geriatrician*:ab,ti OR gerontologist*:ab,ti OR (speech NEAR/3 pathologist*):ab,ti OR nutritionist*:ab,ti OR dietician*:ab,ti OR dietitian*:ab,ti | 581730 |
| #1 | ‘oral assessment*’:ab,ti OR (('mouth hygiene'/exp OR ‘oral health’:ab,ti OR ‘oral care’:ab,ti OR ‘oral hygiene’:ab,ti OR dental:ab,ti) AND ('needs assessment'/exp OR 'nursing assessment'/exp OR 'geriatric assessment'/exp OR assessment*:ab,ti OR assessing:ab,ti OR screening:ab,ti)) | 17549 |

**Search strategy in Cinahl (via EBSCO) 13 November 2017 (read from bottom-up).**

| **Set** | **Search terms** | **Result** |
| --- | --- | --- |
| #4 | (S1 AND S2 AND S3 AND S4) | 91 |
| #3 | ( MH ("Aged+" OR "Aged, 80 and Over" OR "Frail Elderly" OR "Geriatrics" OR "Geriatric Psychiatry" OR "Gerontologic Nursing+" OR "Gerontologic Care" OR "Health Services for the Aged" OR "Dementia" OR "Alzheimer's Disease") ) OR TI ( (elder* OR eldest OR frail* OR geriatri* OR "old age*" OR "oldest old*" OR senior* OR senium OR "very old*" OR septuagenarian* OR octagenarian* OR octogenarian* OR nonagenarian* OR centarian* OR centenarian* OR supercentenarian* OR "older people" OR "older subject*" OR "older patient*" OR "older age*" OR "older adult*" OR "older man" OR "older men" OR "older male" OR "older woman" OR "older women" OR "older female" OR "older population*" OR "older person*" OR alzheimer* OR demented OR dementia OR “cognitive decline” OR “cognitive impairment” OR “nursing home*” OR “assisted living facilit*”) ) OR AB ( (elder* OR eldest OR frail* OR geriatri* OR "old age*" OR "oldest old*" OR senior* OR senium OR "very old*" OR septuagenarian* OR octagenarian* OR octogenarian* OR nonagenarian* OR centarian* OR centenarian* OR supercentenarian* OR "older people" OR "older subject*" OR "older patient*" OR "older age*" OR "older adult*" OR "older man" OR "older men" OR "older male" OR "older woman" OR "older women" OR "older female" OR "older population*" OR "older person*" OR alzheimer* OR demented OR dementia OR “cognitive decline” OR “cognitive impairment” OR “nursing home*” OR “assisted living facilit*”) ) | 488514 |
| #2 | ( MH ("Nurses+" OR "Nursing Assistants" OR "Practical Nurses" OR "Caregivers" OR "Physician Assistants" OR "Speech-Language Pathologists" OR "Allied Health Professions+" OR "Geriatricians" OR "Dietitians") ) OR ( TI ( nurs* OR caregiver* OR carer* OR “care giver*” OR ‘‘informal care*” OR ”community health worker*” OR “physician* assistant*” OR “doctor* assistant*” OR “non-dental professional*” OR “non dental professional*” OR “non-dental staff” OR “non dental staff” OR geriatrician* OR gerontologist* OR speech pathologist* OR nutritionist* OR dietician* OR dietitian*) ) OR ( AB (nurs* OR caregiver* OR carer* OR “care giver*” OR ‘‘informal care*” OR ”community health worker*” OR “physician* assistant*” OR “doctor* assistant*” OR “non-dental professional*” OR “non dental professional*” OR “non-dental staff” OR “non dental staff” OR geriatrician* OR gerontologist* OR speech pathologist* OR nutritionist* OR dietician* OR dietitian*) ) | 509162 |
| #1 | (TI “oral assessment*” OR AB “oral assessment*”) OR ( ((MH "Oral Hygiene+") OR (TI (“oral health” OR “oral care” OR “oral hygiene” OR dental)) OR (AB (“oral health” OR “oral care” OR “oral hygiene” OR dental)) ) AND (MH ("Needs Assessment" OR "Nursing Assessment" OR "Geriatric Assessment+") OR (TI (assessment* OR assessing OR screening)) OR (AB (assessment* OR assessing OR screening))) | 3526 |
